# How Blood Is Purified

**Published:** 1878-05

**Authors:** 


After the air in the lungs has given the finishing stroke, which makes pure and perfect blood out of the heterogeneous mass before described, it is sent back to the great receiving reservoir of the system, the left side of the heart, and is sent by thousands of *distributing* pipes, or blood vessels, to every fibre of the human frame, to be made into flesh, and bone, and joint, and ligament, wherever renovation is needed. And how minutely grand the process. The instant the air meets the impure and imperfect fluid mass in the lungs, it is converted into life, as instantaneous as chrystalization, as quick as the very lightning. This life consists in forming a little boat or cell, like a Nautilus on the sea; in this boat is an atom of life-giving life, which is freighted along the current of the blood, until it arrives at its destined port; the instant of its striking, the vessel is broken, the living atom, as instantaneously as the needle to the armature, bounds to its new home and is a part of the living man, in its turn to die and be washed away to make place for others. How wonderful is our life! How grandly mysterious, and how beautifully wise, is He who made it.

The reader has no doubt felt long ago in this narration how doubly essential to human health is the pure air of heaven, for it alone can purify the blood; it only can make blood out of the nutriment of the system. How infinitely essential, how gloriously useful is PURE AIR AND A PLENTY OF IT, in making the human frame all that it ought to be—all that it was intended to be.

But if the food be imperfect, its nutritive essence must be imperfect, and no air, however pure in quality, or in quantity large, can make a perfect blood out of it. We thus arrive at a sweeping general fact, that in order to have a perfect life-giving blood under the most favorable circumstances, the food we eat must be perfect.

The vegetables we cook must be fresh and perfect of their kind. The meats we consume must be the untainted meat of healthy animals. And both vegetables and meats should be properly and well cooked, and no more.

